# Dental Caries Prevalence and Tooth Loss in Chilean Adult Population: First National Dental Examination Survey

**DOI:** 10.1155/2012/810170

**Published:** 2012-12-18

**Authors:** I. Urzua, C. Mendoza, O. Arteaga, G. Rodríguez, R. Cabello, S. Faleiros, P. Carvajal, A. Muñoz, I. Espinoza, W. Aranda, J. Gamonal

**Affiliations:** ^1^Cariology Area, Department of Restoring Dentistry, Faculty of Dentistry, University of Chile, 8380-492 Santiago de Chile, Chile; ^2^School of Public Health, Faculty of Medicine, University of Chile, 8380-453 Santiago de Chile, Chile; ^3^Periodontal Biology Laboratory, Department of Conservative Dentistry, Faculty of Dentistry, University of Chile, Calle Sergio Livingstone pohlhammer 943, Independencia, 8380-492 Santiago de Chile, Chile; ^4^Public Health Area, Faculty of Dentistry, University of Chile, 8380-492 Santiago de Chile, Chile; ^5^Oral Pathology Area, Faculty of Dentistry, University of Chile, 8380-492 Santiago de Chile, Chile

## Abstract

The purpose of this study was to assess the prevalence of dental caries, tooth loss, and risk factors among adult population of Chile. Furthermore, age, gender, and behavioural specific differences in caries prevalence and tooth loss were examined. A national stratified multistage probabilistic sample design in two-age cohorts was applied to the Chilean population. A sample of 1553 adults, comprising 1088 individuals aged 35–44 and 465 senior individuals aged 65–74, were examined. The DMFT was evaluated following WHO recommendations using diagnostic criteria of caries lesions into dentin. The data were analyzed by univariate and multivariate models using logistic regression analyses. Results showed a mean DMFT of 15.06 in the 35–44-year-old group and of 21.57 in the 65–74 group. Factors related to tooth loss in the 35–44 group through univariate logistic regression were depression (OR 1.9 CI 95% 1.26–2.85), education level <12 years (OR 2.24 CI 95% 1.31–3.73), personal income (OR 1.51 CI 95% 1.04–2.19), and familiar income (OR 2.05 CI 95% 1.34–3.13), and through multivariate logistic regression in the same age group were depression (OR 1.93 CI 95% 1.24–3.0), education level <12 years (OR 1.94 CI 95% 1.2–3.14), and familiar income (OR 1.71 CI 95% 1.09–2.68). Factors related to tooth loss in the 65–74-year-old group through univariate logistic regression were education level <12 years (OR 2.54 CI 95% 1.3–4.96) and personal income (OR 1.66 CI 95% 1.05–2.63), and for multivariate logistic regression in the same age group, it was education level <12 years (OR 2.51 CI 95% 1.21–5.18). In conclusion, adult population in Chile showed a high prevalence of dental caries and tooth loss, as age, education level, personal and familiar incomes, and depression are being the main risk factors.

## 1. Introduction

Dental caries is a disease with both high prevalence and severity in adult worldwide populations. Dental caries affects over half of the population in industrialized countries and since it is a cumulative process, the number of affected individuals increases with ageing [1–4]. Several studies show that dental caries in adult populations affects 5 to 10 teeth per individual, being the most significant cause of tooth loss among adults [5–8]. The damage caused by caries leads to a decrease in quality of life of affected individuals and high economic costs for both individuals and society, which turns this disease into an important public health problem [5–8].

According to the World Health Organization (WHO), chronic diseases associated to poor oral health are increasing in developing countries. More specifically, higher levels of edentulousness, caries and periodontal diseases are mentioned [3]. 

WHO recommends epidemiological studies on population aged 35–44 and 65–74 due to the importance of these age groups in describing and analysing the cumulative damage of caries on people's oral health over the years [9, 10].

Assessing oral health status in these population groups is important to gather relevant information for planning dental care services and also to generate evidence on final outcomes from dental care delivered to people during their entire life cycle [8, 11, 12]. 

In Chile, before the First Chilean National Dental Examination, no previous study included examination by dentists to measure prevalence of caries or tooth loss in adults. Therefore, the resources that are needed to treat affected people remain unknown. Data collected in this study may be useful to build a more complete profile of oral health conditions of the adult population of South America and be a complement to the analysis of global trends of the disease.

The objective of this study is to determine the prevalence of caries and tooth loss among Chilean adult population aged 35–44 and 65–74 years old, using data from the First Chilean National Dental Examination Survey and to identify risk factors associated with tooth loss.

## 2. Material and Methods

### 2.1. Sampling and Sample Sizes

The study population was gathered through the First Chilean National Dental Examination Survey and comprised noninstitutionalized adults aged 35–44 and seniors aged 65–74 years. All individuals lived in one of the 15 administrative regions of Chile, which in turn, constituted a cluster random sample based on region (in this study, the sample only considered urban population). The random sample included 1553 individuals, 1088 adults and 465 seniors, corresponding to age distribution of the Chilean population.

Sampling was based on data from the National Institute of Statistics (NIS). PThe population corresponds to the official 2008 estimates from the 2002 national census, distributed according to age, gender, and region of residency. Sample size calculation was performed estimating a prevalence of 80% who suffer dental caries; the sample size necessary to achieve a 95% (confidence interval: 95%) precision rate, with a 0.02 per cent range of error, was calculated to be 1553 adults (sample size with a statistical weight).

Adults selected to participate in this study were chosen using a multistaged probabilistic sample model that involved all 15 administrative regions in Chile. A sample with statistical weight was used to generate unbiased total variance estimations. Sample statistical weights were adjusted in order to obtain a certain number of individuals according to the referent base population of Chile, considering the differences by gender, age, region location, and city size class. In the first stage, the samples of all fifteen regions (primary sampling units) were obtained from data registered in the NIS. From the same source, we obtained the sample of each regions capital city (second stage). The third stage consisted of the municipalities in order to select the specific houses (fourth stage). All the adults in the sample were proper candidates for examination. In case of refusal to participate, replacements came from the next household belonging to the sex and age group that accepted to participate until completing the number of individuals defined for each specific commune.

More than 40% of the total Chilean population resides in the Metropolitan Region, this region includes the national capital city (Santiago) and 32 municipalities more. The sample in the Metropolitan Region was taken considering 32 districts that were distributed by socioeconomic level according to a random protocol.

The protocol of this study along with the informed consent forms were approved by an independent bioethical committee that belongs to the Faculty of Medicine of the University of Chile, according to the legal regulations requested. The study protocol was previously explained to all patients, and informed consents were signed for each individual prior to entering the study. At the end of the clinical examinations, participants who were diagnosed with pathological conditions were provided with a written report of their condition and were advised to seek oral health care.

### 2.2. Clinical Evaluation, Sociodemographic, and Behavioral Data Collection

The survey comprised a social and health interview and dental examinations. Before clinical examinations in the field, a calibration procedure was carried out by a senior member from the Cariology area of Faculty of Dentistry, University of Chile. The Kappa statistic indexes of the 8 examiners were between 0.80 and 0.90 for intraclass and interclass coefficient. Validity and reliability examinations were carried out during the field period at the beginning, middle and end of the study period. The clinical evaluation was performed in conventional dental clinics that belonged to the Primary Health Care Services near the selected house.

DMFT scores were recorded following WHO recommendations using diagnostic criteria of caries lesions into dentin [9]. An oral health questionnaire was applied by a trained interviewer. Interview time was around 30 minutes and was performed before clinical examination.

The clinical examiners were not aware of the conditions of the individuals involved.

The monthly family income was based on the Chilean minimum wage ($285.000 Chilean currency, about USD 570 per month). Chilean school system and school education was categorized as 12 years or 13 years of schooling. Other variables such as gender, age (35–44 and 65–74-year-old), diabetes, obesity, and depression (self reported) were registered.

## 3. Statistical Analysis

Firstly, for categorical variables a descriptive exploratory analysis of data, including frequency description (absolute values and percentages) was performed; for continuous variables such as age and DMFT both dispersion and central, tendency measures were determined, and confidence intervals were calculated.

Univariate and multivariate regression models using logistic regression analyses were calculated for factors associated with tooth loss.

## 4. Results

Out of the total sample, 18% of individuals were replaced because they were not at home or for refusing to participate in the study.

Table 1 shows the gender distribution for both groups (35–44 and 65–74 year olds) of individuals from all regions of Chile that were included in the study.

For the 35–44 year-olds, in Table 2, data show that the mean of DMFT index was 15.06 (CI 95% 14.70–15.43). In this age group the DMFT index shows statistical differences in gender.

The DMFT index for the 65–74 year-olds was 21.58 (CI 95% 20.86–22.29). In this age group the DMFT index suggest no statistical difference between genders in this age group.

The mean of untreated caries lesions found was 4.2 (CI 95% 4.01–4.47) per individual in the younger group and 1.44 (CI 95% 1.26–1.63) in the older group. We also found that the mean of untreated caries lesions was higher in men in both groups. The mean of filled teeth without caries lesions was 4.9 (CI 4.64–5.19) and 2.66 (CI 2.34–2.99) for the 35–44 and 65–74 year-olds respectively. Although the mean of filled teeth without caries lesions for women was higher than it was for men in both groups, the differences were statistically significant only for the younger group.

The mean of missing teeth due to caries was 5.9 (CI 95% 5.59–6.21) and 17.46 (CI 95%16.59–18.32) for the 35–44 and 65–74 year-olds respectively. Although the mean for women was higher than it was for men in both groups, differences were not statistically significant.

Table 3 shows the distribution of number of teeth present by age group and gender. The data shows differences in number of teeth present between age group. The younger group shows less tooth loss than the older group but gender differences were not statistically significant.

In the 35–44 years old group, univariate logistic regression analysis shows significant association between education level (less than 12 years of education), personal income less than $141.000 Chilean currency (pesos), familiar income less than $285.000 Chilean pesos, self-reported depression and self-reported obesity with the presence of less than 21 teeth. Multivariate logistic regression analysis shows significant association between education level, personal income less than $141.000 Chilean pesos, familiar income less than $285.000 Chilean pesos and self-reported depression with the presence of less than 21 teeth (Table 4).

In the 65–74 year-old group, univariate logistic regression analysis shows significant association between familiar income less than $285.000 Chilean pesos with the presence of less than 21 teeth (Table 5). 

## 5. Discussion

The sample used in this study is representative of the population between 35–44 and 65–74 years old in Chile. This is the first study including these years of age groups at the national level. According to WHO recommendations for collecting epidemiological data, the most important comparable years of age groups are represented by 35–44 and 65–74 years old individuals [9].

In Chile, as in most countries in the world, population is aging. The Chilean population has experienced a process of demographic transition, with an important decrease in fecundity rate and mortality rate in all ages. Life expectancy has raised from 58 years old in the 1960–65 period to 78.12 years old in 2012. Chile is challenged to improve and maintain oral health in adult and senior populations. 

In Chile, oral health policies are hardly focused on populations up to 20 years old. Therefore, the oral health care services for adults are scarce [13].

According to WHO recommendations, oral health surveillance for monitoring adults oral health conditions must be done in the 35 to 44-year-olds. In this group, we can evaluate the effects of dental caries, periodontal diseases, and in general, the effects of oral health care services [9]. On the other hand, 65 to 74-year-olds is an important age group for seniors' oral health monitoring, since population's aging and the increase in life expectancy. The results of this study can be used for oral health care planning and for evaluation of final results of the dental services and care of the elder population [9]. From this point of view, the data collected is highly relevant for the design, implementation, and evaluation of oral health public policies tending to diminish the inequities in health. 

In spite of the importance of epidemiological studies in adults and seniors, only a very reduced number of articles about the dental situation of these years of age groups in national sample from Latin America has been published. These studies show a very high prevalence of caries, affecting 65.3–100% of the mentioned population [14–16].

 The DMFT index values observed in our study (15.06 in 35–44-year-old and 21.57 in 65–74-year-old groups) are higher than those found in more developed countries. For example, in New Zealand, the mean DMFT in 35–44-year-old was 10.0 (95% CI 9.2–10.8) and 24,2 (95% IC: 23,5–24,9) in 65–74-year-old adults [17]. In the second National Survey of Oral Health in China (2002), the mean DMFT in 35–44-year-old adults was 2.1 (DS: 2.8) and 12.4 (SD 17.4) in the 65–74-year-old group [17]. In Spain, the national surveys in the 35–44-year old group made in 1984, 1993, 2000, and 2005 showed a DMFT of 11.6, 10.9, 8.4, and 9.6, respectively, whereas for 65–74-year-old adults, the 3 national surveys in 1993, 2000 and 2005 showed a mean DMFT of 21.16, 18.10, and 16.8, which shows a decrease in the caries experience [18]. 

Only two South American countries have carried out surveys with national samples with oral exam in adults: Colombia (1999) with a DMFT of 15.00 in the 35–44-year-old group and 19.6 in the “older than 55” group [14] and Brazil with a mean DMFT of 20.1 for 35–44-year-olds and 27.3 for the 65–74-year-old group in 2003 and a 16.3 and 27.0 DMFT for the 35–44 and the 65–74 adults, respectively, in 2010 [16]. In Chile, according to our study, we can see a mean DMFT of 15.06 and of 21.57 for the 35–44 and the 65–74 adults, respectively. 

Our findings in terms of caries prevalence are lower than previous studies carried out during the 90s in Chile. Gamonal (1996) found almost 100% of caries experience in low and middle-low socioeconomic level adult populations [19]. We found statistically significant differences between males and female in both years of age groups. Unfortunately, separate figures from previous studies for both men and women are not available for comparisons. This reduction in the damage level of the population's oral health possibly is related to the implementation of a water fluoridation programme in Chile in the mid-1990s and the enhanced conditions in the quality of life in the Chilean population throughout the last years.

The level of edentulism is a good indicator of populations' oral health because the main cause may not only be a carious process, but also many other reasons. In several developed countries, we can see a decrease in the prevalence of edentulism in the new adult cohorts. In the USA, among the young adults group, in the NHANES III 1988–1994, the prevalence of edentulism was 10.8%, and in 1999–2002 it diminished to 7.7%. The level of edentulism of the present study can be seen as an unfavourable situation compared to other countries like Canada, United States, Lithuania, and China [17, 20, 21]. 

Moreira et al. [22] in a systematic literature review from 1986 to 2004 concerning the most prevalent oral problems experienced by elderly Brazilians reported a high percentage of edentulism, with a prevalence between 5.2 and 84%, with the major reported numbers with a prevalence over 60%. Tooth loss has been shown to be associated with oral health impact profile, worse physical functioning, and low perception in quality of life [23, 24]. 

The condition of diabetes, depression, and obesity was declared by the patients during the questionnaire applications, and these conditions were not clinically evaluated. Among the reasons for the increase observed in tooth loss in individuals that declared depression, we found a relation to less personal care, alterations in immune response, and the frequent consumption of medication that affects salivary flow. Depression is also found more frequently in low socioeconomical status individuals [25].

In conclusion, adult population in Chile showed a high prevalence of dental caries and tooth loss, as age, education level, personal and familiar incomes and depression are being the main risk factors. This situation constitutes a great challenge for the health profession considering the increasing of life expectancy of the population.

These observations in caries prevalence in the adult population in Chile suggest that it may be necessary to develop and implement oral health policies taking into account geographical and socioeconomical differences in populations. Because of the lack of epidemiological studies in adults in the South American countries, the data collected in this study may be useful to build a more complete profile of oral health conditions, as well as being a complement to the analysis of the global trends of the disease.

## Figures and Tables

**Table 1:** Characteristics of the sample.

	Age groups (years)				
	35–44		65–74		Total
Female	597	54.87% (95% CI 51.9–57.9)	288	61.94% (95% CI 57.35–66.36)	885
Male	491	45.13% (95% CI 42.14–48.14)	177	38.06% (95% CI 33.63–42.65)	668

Total	1088		465		1553

**Table 2:** DMFT index and its components (mean and 95% confidence interval).

Age groups (years)	Gender	DMFT	D	M	F
	Male	14.10^a^ (13.56–14.64)	4.5 (4.16–4.84)	5.68 (5.24–6.11)	3.91 (3.54–4.28)^b^
35–44	Female	15.86^a^ (15.38–16.33)	4 (3.72–4.35)	6.08 (5.64–6.52)	5.74 (5.35–6.13)^b^
	All	15.06 (14.7–15.43)	4.2 (4.01–4.47)	5.9 (5.59–6.21)	4.9 (4.64–5.19)

	Male	20.41 (19.20–21.62)	1.78 (1.45–2.11)	16.28 (14.88–17.67)	2.34 (1.86–2.82)
65–74	Female	22.29 (21.41–23.17)	1.24 (1.02–1.56)	18.18 (17.09–19.27)	2.86 (2.43–3.3)
	All	21.57 (20.86–22.29)	1.44 (1.26–1.63)	17.46 (16.59–18.32)	2.66 (2.34–2.99)

^a^ *P* < 0.05, ^b^ *P* < 0.05.

**Table 3:** Distribution of number of teeth present in different age groups (% of individuals and 95% confidence interval).

Number of teeth present in mouth	Age groups (years)					
	35–44			65–74		
	All	Female	Male	All	Female	Male
	% (95% CI)	% (95% CI)	% (95% CI)	% (95% CI)	% (95% CI)	% (95% CI)
21 or more teeth	85.66 (83.57–87.74)	85.59 (82.77–88.41)	85.74 (82.64–88.84)	23.87 (19.98–27.75)	21.8 (17.07–26.67)	27.12 (20.53–33.7)
Less than 21 teeth	9.83 (8.06–11.61)	9.54 (7.18–11.9)	10.18 (7.5–12.86)	21.08 (17.35–24.79)	23.26 (18.36–28.16)	17.51 (11.88–23.14)
Less than 15 teeth	4.23 (3.03–5.42)	4.52 (2.85–6.19)	3.86 (2.16–5.58)	43.66 (39.13–48.18)	40.97 (35.27–46.68)	48.02 (40.62–55.42)
Edentulous	0.28 (−0.03–0.58)	0.33 (−0.13–0.8)	0.2 (0.19–0.6)	11.4 (8.49–14.29)	13.89 (9.88–17.9)	7.34 (3.48–11.21)

**Table 4:** Non-adjusted and adjusted odds ratios (ORs) and 95% confidence interval (95% CI) related to tooth loss (the presence of less than 21 teeth in mouth) for the 35–44-year-old group.

Variables	Univariate OR (95% CI)	*P* value	Multivariate OR (95% CI)	*P* value
Education				
>13-years	1.0 (ref)			
<12-years	**2.13** **(1.32–3.73)**	**0.002**	**1.83** **(1.2–2.81)**	**0.006**
Personal income				
>$142.000	1.0 (ref)		1.0 (ref)	
<$141.000^&^	**1.65** **(1.15–2.38)**	**0.007**	1.34 (0.89–2.0)	0.16
Familiar income				
>$286.000	1.0 (ref)		1.0 (ref)	
<$285.000*	**2.28** **(1.5–3.46)**	<**0.0001**	**1.94** **(1.24–3.04)**	**0.004**
Diabetes				
No	1.0 (ref)		1.0 (ref)	
Yes	1.5 (0.78–2.89)	0.22	1.14 (0.56–2.32)	0.71
Depression				
No	1.0 (ref)		1.0 (ref)	
Yes	**1.89** **(1.27–2.81)**	**0.002**	**1.93** **(1.24–3.0)**	**0.003**
Obesity				
No	1.0 (ref)		1.0 (ref)	
Yes	**1.67** **(1.05–2.63)**	**0.03**	1.54 (0.94–2.52)	0.085

^&^$141.000 Chilean pesos, about USD 271 per month.

*$285.000 Chilean pesos, about USD 570 per month.

Statistically significant associations in boldvalues.

**Table 5:** Nonadjusted and adjusted odds ratios (ORs) and 95% confidence interval (95% CI) related to tooth loss (the presence of less than 21 teeth in mouth) for the 65–74-year-old group.

Variables	Univariate OR (95% CI)	*P* value	Multivariate OR (95% CI)	*P* value
Education				
>13-years	1.0 (ref)			
<12-years	1.97 (0.98–3.96)	0.056	1.96 (0.93–4.16)	0.07
Personal income				
>$142.000	1.0 (ref)		1.0 (ref)	
<$141.000^&^	1.55 (0.95–2.53)	0.076	1.24 (0.7–2.22)	0.45
Familiar income				
>$286.000	1.0 (ref)		1.0 (ref)	
<$285.000*	**1.66** **(1.02–2.69)**	**0.038**	1.05 (0.62–1.8)	0.83
Diabetes				
No	1.0 (ref)		1.0 (ref)	
Yes	1.27 (0.75–2.16)	0.361	1.06 (0.61–1.84)	0.83
Depression				
No	1.0 (ref)		1.0 (ref)	
Yes	1.35 (0.79–2.31)	0.26	1.12 (0.63–2.0)	0.7
Obesity				
No	1.0 (ref)		1.0 (ref)	
Yes	1.99 (0.95–4.19)	0.068	1.84 (0.85–3.99)	0.12

^&^$141.000 Chilean pesos, about USD 271 per month.

*$285.000 Chilean pesos, about USD 570 per month.

Statistically significant associations in bold values.
